# Analysis of variable number of tandem repeats of ‘*Candidatus* Liberibacter asiaticus’ using CLIBASIA_01645 locus

**DOI:** 10.3389/fmicb.2026.1854860

**Published:** 2026-10-06

**Authors:** Ashish Warghane, Rashmi Saini, Nisarg Simariya, Yash Solanki, Gaurav Shrimali, Rajeshkumar G Chaudhary, Vaibhav Bhatt

**Affiliations:** 1 GTU-School of Applied Sciences and Technology (SAST), Gujarat Technological University, Ahmedabad, Gujarat, India; 2 Department of Zoology, Gargi College, University of Delhi, New Delhi, India

**Keywords:** Asian citrus psyllid (ACP), *Candidatus* Liberibacter asiaticus (*C*Las), Huanglongbing (HLB), molecular variability, tandem repeat number (TRN)

## Abstract

‘*Candidatus* Liberibacter asiaticus’ (*C*Las) is a phloem-limited bacterium that causes Huanglongbing (HLB), the devastating citrus greening disease that has led to billions of dollars in losses throughout global citrus production. There is very little epidemiological data available regarding fine-scale molecular variability of *C*Las populations in different regions of the world or their distribution across various geographical landscapes. Here, we present an assessment of *C*Las genetic diversity using a hypervariable variable number tandem repeat (VNTR) marker (AGACACA) at the CLIBASIA_01645 locus of 344 genomic sequence data obtained from GenBank from five regions of the world (India-177, China-113, Saudi Arabia-37, Bhutan-10, and Nepal-7). The findings revealed high evolutionary plasticity and non-random distribution of TRN within four distinct classes, significantly deviating from a uniform distribution (*p* < 0.05). The analyzed populations were stratified into four distinct classes: Class I (TRN ≤ 5), Class II (TRN > 5 ≤ 10), Class III (TRN > 10 ≤ 15), and Class IV (TRN > 15). The predominant type of subpopulation for all field samples was intermediate variants, with the highest frequencies observed for TRN6 (25%) and TRN7 (17.15%), possibly indicating genomic stability and/or adaptive advantages of specific tandem repeats. On the other hand, lower frequency rare variants (e.g., TRN17, TRN18, and TRN20 at 0.29%) represent some form of continued low frequency micro evolutional replication slippage and corresponding absence of some individual TRs indicates evidence of local purging forces. Crucially, the Indian *C*Las populations exhibited significantly greater densities of molecular diversity than populations from China, Saudi, Bhutan, and Nepal examined in this study. The distinct TRN signature profiles of these populations’ subpopulations exhibit strong geographic correlation with ecological regions, reinforcing the CLIBASIA_01645 locus as a strong marker for epidemiological surveillance for tracking global strain movement to develop target-specific HLB management programs.

## Graphical abstract

**Figure:**
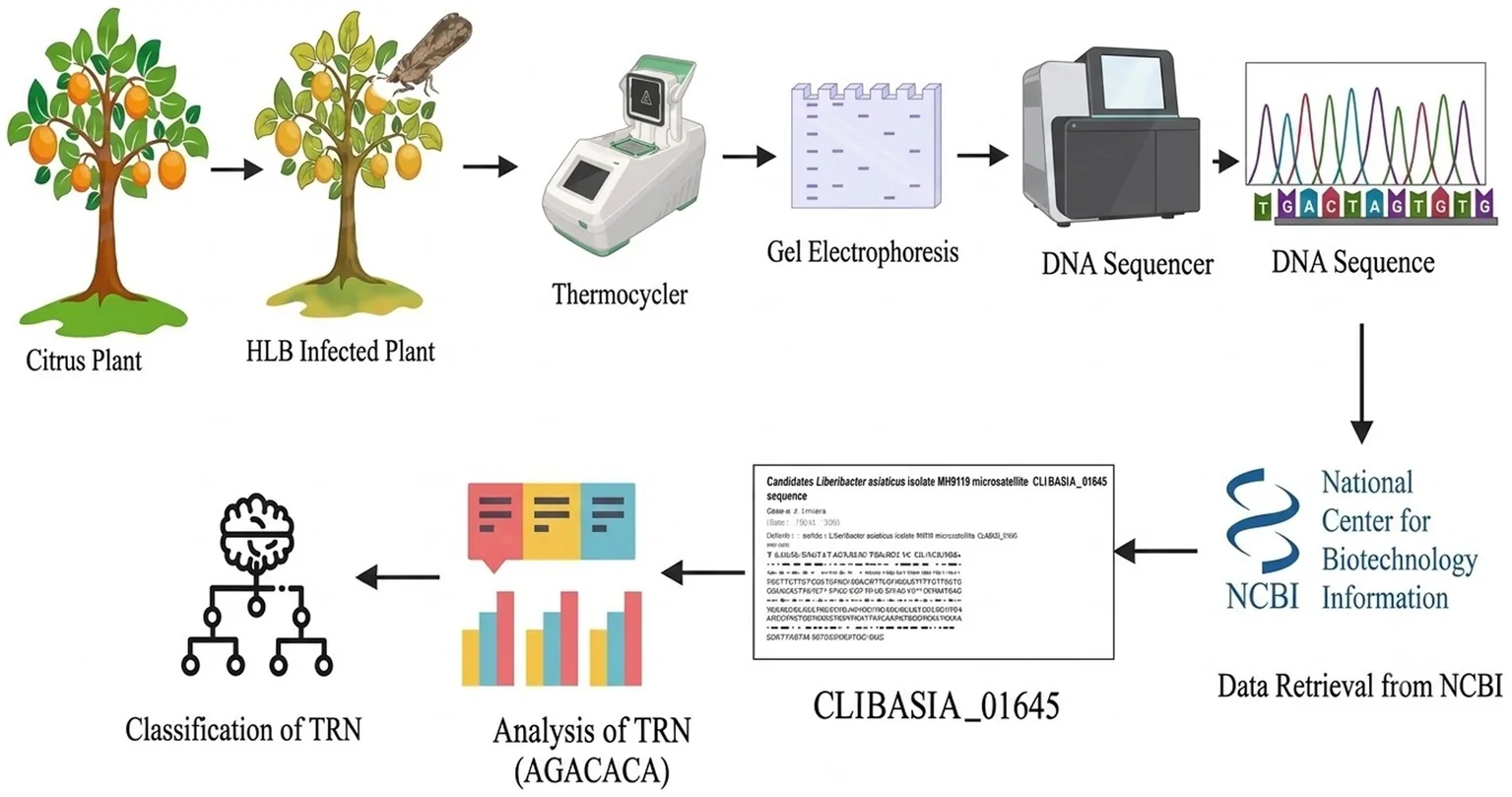
Workflow for the identification and characterization of tandem repeat number.

## Highlights

- First comprehensive analysis of *C*Las variability using VNTRs, incorporating sequence data from major citrus-growing countries in Asia and the Middle East.
- Indian *C*Las populations exhibited significantly higher VNTR-based diversity compared to those from China, and Bhutan.
- Four distinct classes of CLas were identified based on tandem repeat number (TRN): Class I (TRN ≤ 5), Class II (TRN 6–10), Class III (TRN 11–15), and Class IV (TRN > 15).
- Novel insights into the molecular epidemiology of *C*Las and its implications for citrus greening disease management and evolution.

## Introduction

Citrus greening, also known as Huanglongbing (HLB), is a devastating disease that affects citrus plants worldwide (Da Silva et al., 2019; Ghosh et al., 2015). This disease is caused by ‘*Candidatus* Liberibacter asiaticus’ (*C*Las), a phloem-limited bacterium that is transmitted by the Asian citrus psyllid (Warghane et al., 2017; Coletta-Filho et al., 2004). Citrus trees that were susceptible to HLB would exhibit stunted yellow shoots, poor foliation, inward leaf curl with vein corking, and even blotchy mottles on the leaves. Eventually leading to tree decline and death (Gottwald, 2010; Bastianel et al., 2005). *C*Las was first discovered in Asia in 1919 and later found in other citrus-producing regions worldwide. *C*Las is a gram-negative bacterium that is known to be present in various citrus-growing regions across the world, including Asia, Africa, and South America (Gottwald, 2010). The economic impact of this disease on citrus production is significant, with losses estimated to be in the billions of dollars annually. HLB has significantly impacted the citrus industry, causing a drop in citrus fruit yield and quality (Spreen et al., 2020). Three gram-negative, *Candidatus* Liberibacter are linked to HLB. The primary citrus growing regions have a high prevalence of *C*Las, but ‘*Candidatus* Liberibacter africanus’ and *‘Candidatus* Liberibacter americanus’ are only locally prevalent. Citrus varieties have been found to tolerate HLB in different ways, despite the absence of a genus-wide inherent immunity to the disease.

A comprehensive understanding of the genetic diversity and evolutionary dynamics of *C*Las strains are fundamental for devising effective strategies to manage Huanglongbing (HLB) disease in citrus. Although early research largely focused on whole-genome characterization of *C*Las, recent years have seen growing emphasis on specific genetic markers, particularly microsatellites and variable number tandem repeats (VNTRs), for fine-scale population analyses. Microsatellites, which are short repetitive DNA sequences, are ubiquitous in bacterial genomes and serve as robust indicators of genetic variation (Katoh et al., 2011). VNTRs represent a subset of microsatellites where the number of repeat units varies among bacterial strains, making them valuable for molecular typing, population structure analysis, and investigating evolutionary dynamics (Katoh et al., 2011). The CLIBASIA_01645 locus was selected because it is one of the most informative VNTR markers for CLas population studies. Previous investigations have demonstrated that this locus possesses high allelic diversity and excellent discriminatory power for differentiating geographically distinct CLas populations, making it a reliable marker for studying genetic diversity and epidemiological relationships.

A unique genomic region of *C*Las exhibiting locus mosaicism was recently characterized, revealing that the isolates in China can be classified into high and low altitude groups (Wang et al., 2012). The availability of the complete genome sequence (1.23 Mb) of the pathogenic *C*Las psy62 (Duan et al., 2009) facilitated diversity analysis of bacterial populations based on tandem repeat numbers (TRN) at specific genomic loci. Simple sequence repeats (SSRs), also known as microsatellites, are tandem repeats with motif lengths of 2 bp or more and have been attributed to genetic variability caused by slipped-strand mispairing, instability of polynucleotide tracts such as poly(G-T), and recombination between homologous repeats (Levinson and Gutman, 1987). SSR markers, particularly variable number tandem repeats (VNTRs), have been effectively used in strain differentiation and subtyping of bacteria including *Xylella fastidiosa*, the causal agent of variegated chlorosis in citrus and Pierce’s disease in grapevines (Lin et al., 2005; Katoh et al., 2011; Islam et al., 2012). Islam et al. (2012) developed microsatellite markers and distinguished three major genetic groups of *C*Las worldwide. Chen et al. (2010) analyzed TRN variation at the CLIBASIA_01645 loci in strains from Guangdong, China, and Florida, United States, reporting that 84.5% of Florida strains exhibited TRN = 5, while 47.6% of Guangdong strains had TRN = 7. Katoh et al. (2011) identified four tandem repeat loci with high sensitivity for detecting diversity among *C*Las isolates from Japan, Taiwan, and Indonesia, with locus CLIBASIA_01645 (also known as locus 005) showing the highest Nei’s diversity value (Singh et al., 2019). Deng et al. (2014) conducted double-locus analyses based on TRN and single nucleotide polymorphisms (SNPs) to evaluate genomic variation in ‘*Candidatus* Liberibacter asiaticus’ populations. HLB also referred to as citrus dieback in India since the eighteenth century, was initially attributed to soil disorders, nutrient deficiencies, fungi, and viruses (Fraser and Singh, 1968; Gao et al., 2022). Formal evidence of HLB in India was established in 1967 following successful transmission by the insect vector citrus psylla (*Diaphorina citri*) (Hall et al., 2013; Bové, 2006). Since then, HLB has been widely reported across Indian citrus growing states including Maharashtra, Andhra Pradesh, Punjab, Tamil Nadu, West Bengal (Darjeeling Hills), Bihar, and northeastern states proximal to China, Nepal, and Bhutan (Bové, 2006; Das, 2008). Despite this, biological and genomic data on *C*Las diversity among Indian regions remain limited. Leveraging the success of differentiating *C*Las populations at the CLIBASIA_01645 locus, the present study focuses on assessing genetic diversity in five different counties populations infecting various citrus cultivars by analyzing variability in the (AGACACA) tandem repeat motif at the same locus.

The assessment of VNTR profiles in *C*Las strains collected from different geographic regions enables researchers to gain critical insights into genetic diversity, evolutionary history, and the geographic spread of this pathogen. Profiling these loci has enhanced the understanding of *C*Las population structure, including global strain migration and associations between particular genotypes and pathogen aggressiveness (Ghosh et al., 2015). One of the significant challenges in HLB management is the extensive genetic diversity exhibited by *C*Las across numerous countries, with VNTR analysis consistently uncovering substantial genomic variability linked to biology and pathogenic traits (Zheng et al., 2021). For instance, VNTR analysis performed in Florida (Chen et al., 2010) revealed multiple *C*Las genotypes, underscoring population diversity within this region, while studies such as (Jagoueix et al., 1996) demonstrated that VNTRs can also differentiate closely related species causing citrus greening in distinct global regions. This approach not only underscores the utility of VNTRs as reliable markers for epidemiological surveillance but also facilitates phylogenetic and evolutionary inferences. The present study focuses on the VNTR locus CLIBASIA_01645, drawing upon VNTR analysis from 344 *C*Las DNA samples gathered from major citrus-growing countries of Asia and middle east.

## Materials and methods

### Data collection and sequence retrieval

To facilitate a comprehensive analysis of *C*Las genetic diversity, a curated sequence dataset was assembled from multiple publicly available resources, with primary emphasis on the National Centre for Biotechnology Information (NCBI), database. Sequence selection prioritized representative geographic distribution, covering both complete and partial genome sequences to maximize diversity and analytical power. Metadata accompanying each accession, including the strain’s geographic origin were meticulously cataloged to enable robust correlation analyses. The resulting dataset provided a representative cross-section of *C*Las genomic diversity, serving as a foundation for further evolutionary and population genetic investigations (Supplementary Table 1).

### VNTR analysis

The collected *C*Las DNA sequences underwent an analysis to identify and investigate the presence and distribution of VNTRs. To achieve this, the RepeatMasker software tool was utilized (Tarailo-Graovac and Chen, 2009). RepeatMasker (version 4.1.6) is widely employed to identify and mask repetitive regions within genome sequences, ensuring accurate identification of unique VNTR regions and preventing redundant analysis. This step helped to focus on the target VNTR regions of interest within the genome. Even though the number of VNTR present in each sequence were analyzed manually using MS-word.

### Identification of sequence analysis and BLAST

To discover additional relevant sequences, the BLAST (Basic Local Alignment Search Tool) platform is widely used for comparative sequence analysis and identification of homologous regions (Altschul et al., 1990). By inputting VNTR or other target sequences from *C*Las into BLAST using databases such as NCBI’s non-redundant nucleotide collection it is possible to retrieve more sequences with high similarity to the query (*E*-Value −0.0, Percent Similarity −95 to 100%), including those from diverse strains or related species. This methodology not only enhances sequence discovery but also supports in-depth comparative TRN distribution, further expanding the dataset for evolutionary and functional analyses of *C*Las (Figure 1).

**Figure 1:**
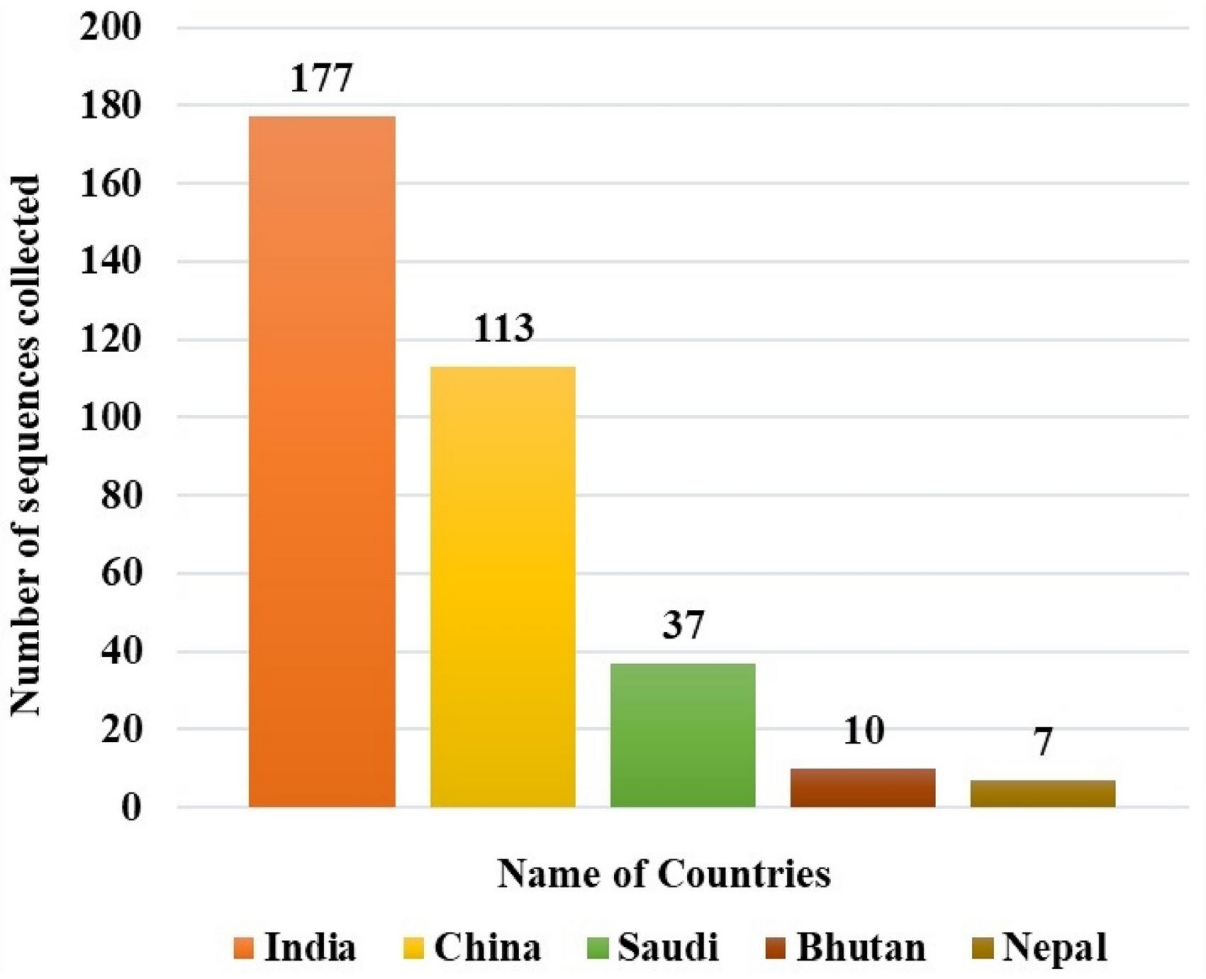
The geographical coverage and sampling density across the study area.

### Determination of TRNs at VNTR locus

The number of tandem repeats (TRNs) at the VNTR locus was determined for each *Candidatus* Liberibacter asiaticus isolate CLIBASIA_01645 region sequence analysis. Based on the TRN copy number, isolates were classified into four groups according to the criteria described by Ghosh et al. (2015): Class I (TRNs ≤ 5), Class II (TRNs > 5 to ≤ 10), Class III (TRNs > 10 to ≤ 15), and Class IV (TRNs > 15). This classification was used to assess the genetic diversity and distribution of *Candidatus* Liberibacter asiaticus populations among the sampled citrus-growing regions and to facilitate comparison with previously reported VNTR genotypes.

## Results

### Analysis of samples collected from different countries

In the present study, a total of 344 *C*Las sequences were collected from five distinct countries, providing a broad representation for comparative genomic analyses. The geographic breakdown of the dataset included 177 sequences from India, 113 from China, 37 from Saudi Arabia, 10 from Bhutan, and 7 from Nepal (Figure 2). This diverse sampling facilitated robust investigations into the genetic variability and population structure of *C*Las strains across key citrus-producing regions in Asia and the Middle East.

**Figure 2:**
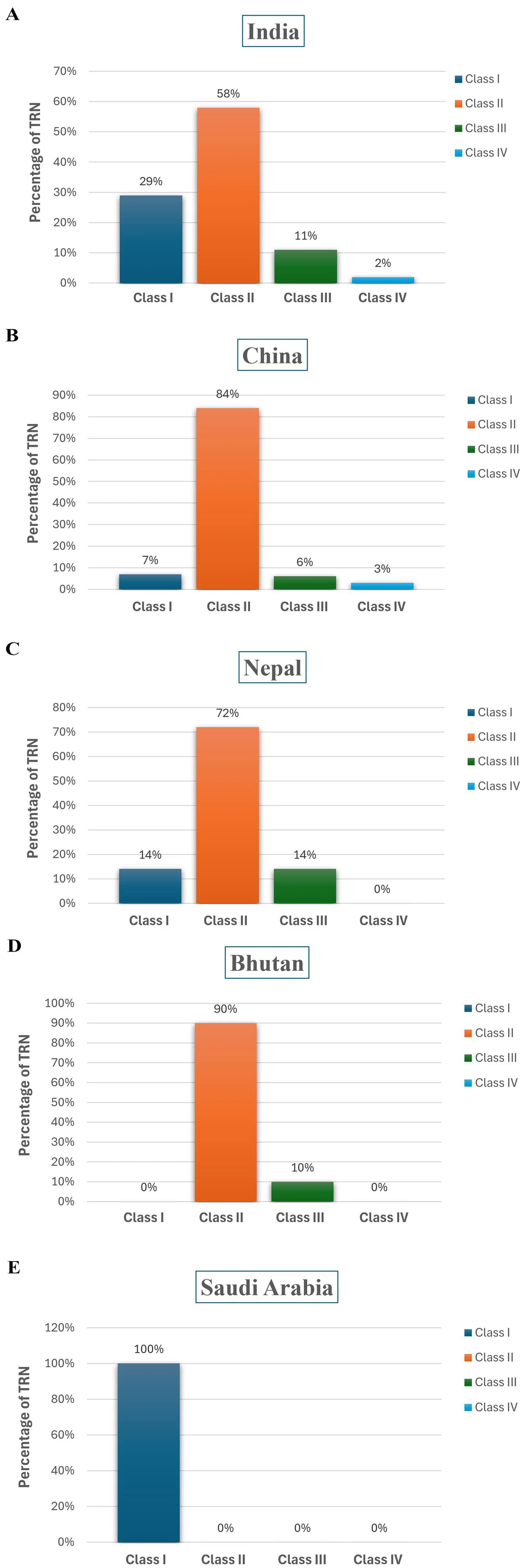
Histograms representing the occurrence of Tandem Repeat Number in the respective countries. **(A)** India, **(B)** China, **(C)** Nepal, **(D)** Bhutan, and **(E)** Saudi Arabia.

### Distribution and percentages of TRN

Based on the TRN classification criteria, the population of *C*Las strains were divided into four distinct classes for further evolutionary and population analysis (Ghosh et al., 2015). Class I (TRNs ≤ 5) comprised strains exhibiting low repeat numbers, which were found at relatively low frequency within the examined cohort, suggesting potential constraints in adaptability or recent evolutionary origin. Class II (TRN > 5 but ≤10) represented a significant proportion of the population and included strains with intermediate repeat numbers, reflecting a balance between mutational events and selective pressures within diverse ecological niches. Class III (TRNs > 10 but ≤15) encompassed strains with higher repeat numbers, pointing toward active microevolution and possible adaptation to varied environmental conditions or host interactions. Finally, Class IV (TRNs > 15) contained a minority of highly repetitive genotypes, which may represent either specialized adaptations or the results of rapid, recent mutational events within specific subpopulations.

This classification revealed distinct patterns in TRN distribution, with Class II and Class III strains predominating, consistent with previous studies demonstrating a tendency for mid-range repeat variants to emerge and persist in field populations. The evolutionary dynamics underlying this distribution are attributed to the high mutability of VNTR loci, enabling quick genomic responses to host defense mechanisms and environmental fluctuations. Furthermore, the observed diversity among classes emphasizes the evolutionary plasticity of *C*Las across geographical regions, contributing to the bacterium’s success as a widespread citrus pathogen (Lin et al., 2005). Importantly, the stratification of strains by TRN class provides valuable markers for population tracking, epidemiological studies, and exploring possible genotype classification.

### Analysis of TRN at the CLIBASIA_01645 locus

Analysis of the tandem repeat numbers (TRN) at the CLIBASIA_01645 locus across *C*Las strains revealed a broad distribution of repeat variants, highlighting notable genetic diversity within the population. The most abundant repeat numbers were TRN6 (25%) and TRN7 (17.15%), followed by TRN5 (13.9%) and TRN4 (11.33%), while lower frequencies were observed for TRNs such as TRN2, TRN3, TRN8, and others, and several repeat types (TRN1, TRN14, TRN15, TRN19) were not detected in the study cohort. The predominance of certain repeat numbers, particularly in the mid-range (TRN4–TRN8), mirrors findings from other regional studies, suggesting that Class II genotypes (intermediate TRN values) are widely distributed and frequently arise within populations due to their potential adaptive advantages (Figure 3).

**Figure 3:**
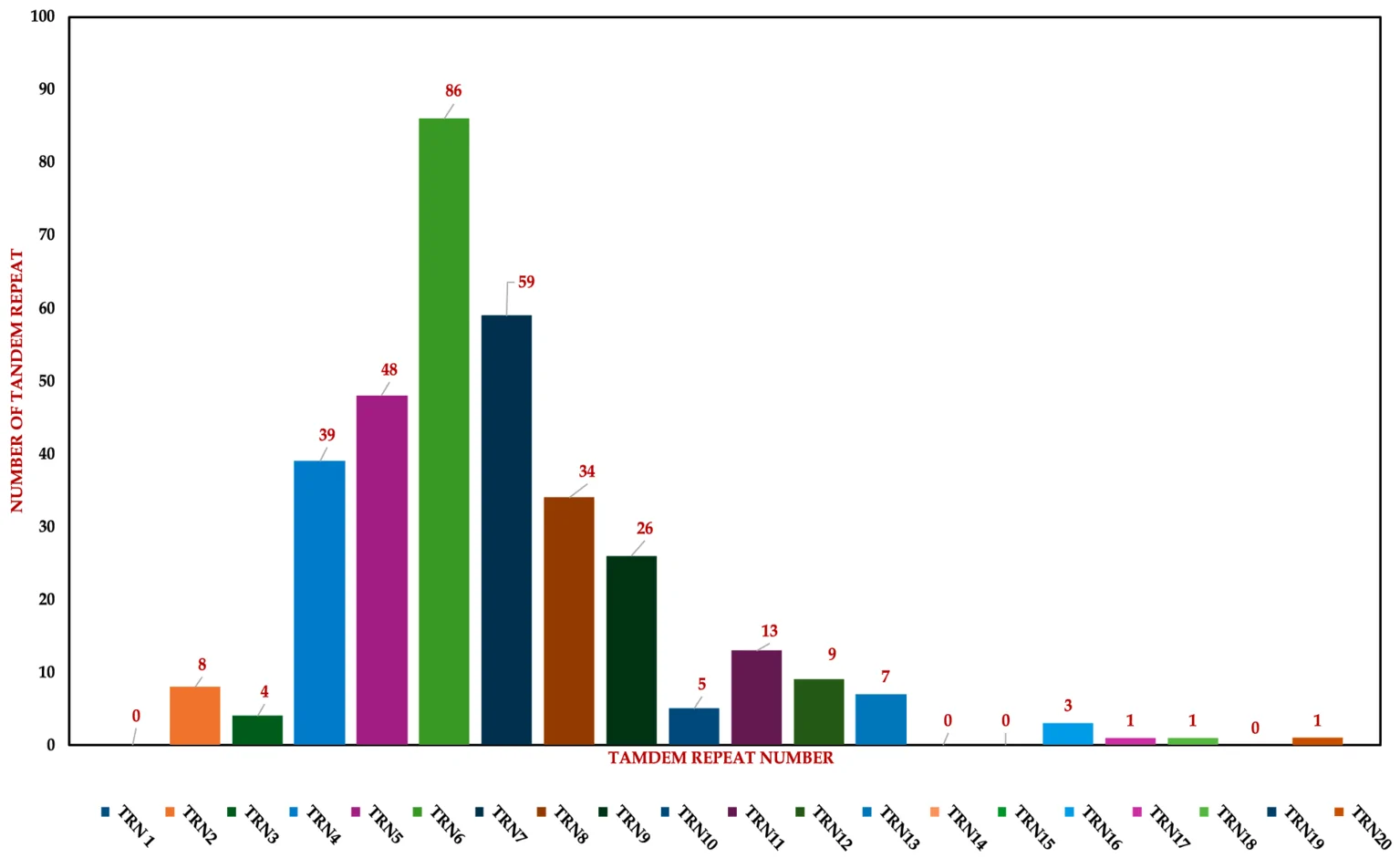
The frequency and pattern of tandem repeat occurrence across the analyzed samples.

To investigate whether geographic origin influenced TRN variation, isolates were grouped according to their country of origin and compared based on the distribution of TRN classes. The frequencies of TRN classes differed significantly among geographic populations (Chi-square test, *p* < 0.05), indicating that TRN distribution is not random but associated with geographical origin. Indian isolates exhibited the greatest diversity, encompassing all four TRN classes, whereas Chinese isolates were dominated by Class II genotypes (primarily TRN6 and TRN7). Saudi Arabian isolates were largely restricted to Class I genotypes, while Bhutanese and Nepalese populations displayed intermediate diversity. These findings support the presence of geographic structuring in *C*Las populations, similar to previous observations reported by Chen et al. (2010).

To evaluate the distribution pattern of tandem repeats across geographic populations, a Chi-square test of independence was performed. The analysis revealed a statistically significant association between TRN class frequency and geographical origin (*χ*^2^ = 284.15, df = 12, *p* < 0.0001), confirming non-random geographic structuring.

## Discussion

Tandem repeats in the genome of *C*Las are short, recurring sequences of DNA that play a key role in assessing the genetic diversity of this citrus pathogen (Katoh et al., 2011). These tandem repeats, often referred to as variable-number tandem repeats (VNTRs) or simple sequence repeats (SSRs), differ in their copy number among *C*Las isolates and are used for molecular typing and population genetics studies (Zheng et al., 2021). Genomic analyses have identified multiple SSR loci in the *C*Las genome, with repeat copy numbers varying widely among strains, ranging from as few as zero to over 20 copies in some loci. VNTR markers allow differentiation of pathogen populations and track the spread of specific haplotypes in newly invaded areas, such as different regions of Asia and America (Deng et al., 2014). Studies have shown that the number of repeats at certain loci remains stable when the bacterium is transmitted between hosts, but some loci display minor variations, which contribute to the genetic signature profiles unique to each population (Saberi et al., 2024; Schlötterer, 2004). Thus, analysis of tandem repeats serves as an important approach for understanding the epidemiology, population structure, and evolutionary adaptation of *C*Las in citrus groves worldwide.

The sequence analysis of the identified VNTR regions, coupled with BLAST searches against NCBI databases, confirmed sequence identity across retrieved *C*Las strains (98–100% identity, E-value = 0.0), validating the accuracy and integrity of the CLIBASIA_01645 locus sequences retrieved for this study. The findings of this study significantly contribute to the existing knowledge on *C*Las by providing a comprehensive understanding of its genetic diversity, population dynamics, and evolutionary characteristics. The results enhance our ability to develop effective disease management strategies by considering the geographic distribution of VNTR profiles, identifying potential targets for control measures, and understanding the adaptive mechanisms of *C*Las.

The observed classification of *C*Las strains into four TRN-based classes reveals important evolutionary and ecological insights. Class I, comprising strains with TRNs ≤ 5, is less frequent, suggesting limited adaptability or recent emergence. Classes II (TRN > 5 but ≤ 10) and III (TRNs > 10 but ≤ 15) dominate the population, reflecting a dynamic equilibrium between mutation and selection pressures shaping strain diversity across environments. The presence of Class IV strains (TRNs > 15), though minor, indicates ongoing rapid genomic changes or niche specialization within subpopulations (Chen et al., 2010). The predominance of mid-range TRNs (particularly TRN6 and TRN7) highlights the selective stability possibly conferred by these repeat numbers, consistent with their prevalence in diverse geographies. This variability is likely driven by the rapid mutation of tandem repeats via slipped-strand mispairing, contributing to the bacterium’s evolutionary plasticity. The absence of some TRNs supports the influence of purifying selection or local extinctions, while rare repeats may represent ongoing mutations at low frequencies. These TRN patterns provide vital molecular markers for population tracking, elucidating genotype–phenotype relationships and informing management strategies against HLB. Overall, the data substantiate the critical role of TRN diversity in *C*Las adaptability and pathogenic success, underpinning the bacterium’s widespread impact on citrus agriculture (Ghosh et al., 2015).

This pronounced variability likely reflects the evolutionary dynamics of *C*Las, as tandem repeat regions mutate rapidly via slip-strand mispairing during DNA replication, providing a substrate for quick adaptation to environmental pressures and host defenses. The frequent detection of TRN6 and TRN7 genotypes across different geographical areas, and their higher representation among strains, points to a selective advantage or stability in the bacterial genome. Furthermore, the absence of certain repeat types could indicate purifying selection or local extinction, while the persistence of rare variants (such as TRN17, TRN18, and TRN20 at 0.29%) suggests ongoing low-frequency mutational events.

To analyze the given tandem repeat number (TRN) data, a suitable approach is to perform descriptive statistics such as the Chi-square test, to determine whether the observed distribution of TRN frequencies significantly deviates from a uniform or expected distribution. The Chi-square test will likely indicate a statistically significant deviation from a uniform distribution, given the uneven distribution of the TRN counts. This means the null hypothesis of equal frequency of all TRNs can be rejected. The observed frequencies show that certain repeats occur far more frequently than others, while some repeats are absent. This non-random distribution suggests that evolutionary forces such as selection, genetic drift, or mutation bias are shaping the population structure of *C*Las.

A Chi-square test of goodness-of-fit confirmed that the observed frequency distribution of TRN values significantly deviated from a uniform distribution (*χ*^2^ = 532.74, df = 19, *p* < 0.0001). This non-random distribution highlights that specific intermediate repeats (e.g., TRN6 and TRN7) predominate, pointing toward evolutionary mechanisms such as replication slippage, selection, or genomic stability favoring specific repeat copy numbers.

Interestingly, no isolates carrying TRN1, TRN14, TRN15, or TRN19 were identified among the 344 *‘Candidatus Liberibacter asiaticus’* genomes analyzed in this study. The absence of these TRN classes suggests that they may represent rare or that they have been reduced through evolutionary processes such as genetic drift or selection. Alternatively, their absence may reflect the limited representation of certain geographic population in the currently available genome database. Further studies incorporating a broader range of isolates from diverse citrus-growing regions will be valuable to determine the distribution and evolutionary significance of these uncommon TRN genotypes.

Although the present study did not specifically reanalyze the original Guangdong isolates reported by Chen et al. (2010), the broader dataset of 113 Chinese genomes showed a comparable pattern in which intermediate TRN genotypes, particularly TRN6 and TRN7, predominated. The increased frequency of TRN6 observed in our study is likely attributable to the inclusion of isolates from multiple provinces and recently available genome sequences, thereby extending the geographic and temporal scope of the earlier investigation. The VNTR locus CLIBASIA_01645 has been validated primarily for population genetic studies of *C*Las and should be regarded as a strain-level epidemiological marker within this species. Its utility for differentiating other *Candidatus liberibacter* species remains to be investigated.

The BLAST analysis was performed primarily to verify the identity and integrity of the retrieved *C*Las sequences used in this study. Since comparative analyses with other *Candidatus Liberibacter* species were not conducted, no conclusions regarding conservation of the CLIBASIA_01645 locus or repeat expansion across related species can be drawn. Future comparative genomic studies may help determine whether this VNTR locus and its repeat variability are unique to *C*Las or shared among other members of the genus.

Importantly, variation in the TRN profiles was found to correlate with the ecological diversity of citrus growing regions and to be associated with specific evolutionary trajectories in the population, supporting the hypothesis that tandem repeat diversity contributes to the bacterium’s adaptability and potential for variation in pathogenicity or virulence (Duan et al., 2009). Thus, the observed spectrum of TRN frequencies underscores the role of micro evolutionary processes in shaping the genetic landscape of *C*Las and highlights the utility of TRN markers for dissecting population structure, migration, and adaptation in this globally important plant pathogen. Through this comprehensive analysis, evolutionary patterns between global *C*Las strains are explored, and potential correlations between VNTR genotypes is investigated (Wang et al., 2012). Such studies have the potential to significantly advance our understanding of *C*Las evolution and inform the deployment of better-informed HLB disease management strategies.

## Conclusion

The *C*Las is a highly destructive bacterium responsible for citrus greening disease, which has caused severe economic losses to the global citrus industry. VNTR analysis has emerged as a powerful tool for investigating the genetic diversity of *C*Las, facilitating a better understanding of the pathogen’s biology, evolution, and spread. The geographic distribution and genetic variability of *C*Las strains underscore the urgent need for continued research to inform effective disease management and control strategies. VNTR profiling helps in tracking pathogen dissemination. Continued exploration of VNTR markers will advance our knowledge genetic diversity and evolutionary dynamics, ultimately contributing to the development of targeted, sustainable control measures to mitigate the devastating impact of citrus greening worldwide.

In conclusion, our study demonstrates that VNTR profiling is a highly useful and accessible tool for evaluating genetic diversity, resolving population structure, and supporting epidemiological surveillance. While our current data do not establish direct functional links between specific profiles and differences in virulence or pathogenicity, they provide the essential baseline genetic framework. Future functional and genomic investigations will be required to determine if these specific VNTR lineages correlate with distinct pathogenic traits or therapeutic targets.

## Data Availability

The data that support the findings of this study are available on request.
