# Supplementary material for: Analysis of variable number of tandem repeats of ‘Candidatus Liberibacter asiaticus’ using CLIBASIA_01645 locus
Source: Front Microbiol. 2026 Oct 6;17:1854860. doi: 10.3389/fmicb.2026.1854860 (PMC13681827; doi:10.3389/fmicb.2026.1854860)
Supplement: Supplementary file 1 [file table_1.docx]

**Supplementary Table 1:** Variable Numbers of Tandem Repeat (VNTR) in CLIBASIA_01645 locus of ‘*Candidatus* Liberibacter asiaticus’ population from different countries and its details.

| **S No.** | **Sample Name** | **Accession No.** | **Location** | | **TRN** | **CLASS** |
| --- | --- | --- | --- | --- | --- | --- |
| 1. | SK-16 | KJ885243 | | India | 5 | I |
| 2. | SK-14 | KJ885242 | | India | 5 | I |
| 3. | SK-13 | KJ885241 | | India | 13 | III |
| 4. | M-4 | KJ885240 | | India | 6 | II |
| 5. | SK-6 | KJ885229 | | India | 8 | II |
| 6. | M-2 | KJ885228 | | India | 4 | I |
| 7. | M-5 | KJ885227 | | India | 7 | II |
| 8. | AK13-Mo | KJ885208 | | India | 6 | II |
| 9. | AK12-Mo | KJ885207 | | India | 7 | II |
| 10. | AK11-Mo | KJ885206 | | India | 13 | III |
| 11. | AK10-Mo | KJ885205 | | India | 7 | II |
| 12. | AK9-Mo | KJ885204 | | India | 7 | II |
| 13. | AK8-Mo | KJ885203 | | India | 7 | II |
| 14. | Ak7-KhM | KJ885202 | | India | 7 | II |
| 15. | Ak5-KhM | KJ885201 | | India | 7 | II |
| 16. | Ak4-KhM | KJ885200 | | India | 7 | II |
| 17. | Ak3-KhM | KJ885199 | | India | 8 | II |
| 18. | Ak2-Khm | KJ885198 | | India | 8 | II |
| 19. | Ak2-Khm | KJ885197 | | India | 7 | II |
| 20. | KhM-vii | KJ885196 | | India | 6 | II |
| 21. | KhM-i | KJ885195 | | India | 9 | II |
| 22. | AsL- i microsatellite | KJ885194 | | India | 7 | II |
| 23. | Mo-I microsatellite | KJ885193 | | India | 8 | II |
| 24. | Mo-I microsatellite | KJ885192 | | India | 7 | II |
| 25. | KhM-iii microsatellite | KJ885191 | | India | 7 | II |
| 26. | Kal-12b microsatellite | KJ921631 | | India | 8 | II |
| 27. | Kal-11b microsatellite | KJ921630 | | India | 5 | I |
| 28. | Kal-10b microsatellite | KJ921629 | | India | 4 | I |
| 29. | Kal-10a microsatellite | KJ921628 | | India | 4 | I |
| 30. | Kal-5 microsatellite | KJ921627 | | India | 5 | I |
| 31. | Kal-4 micsat | KJ921626 | | India | 6 | II |
| 32. | Kal-3 microsatellite | KJ921625 | | India | 7 | II |
| 33. | CH-U6 microsatellite | KJ885223 | | India | 6 | II |
| 34. | CH-CR5 microsatellite | KJ885222 | | India | 5 | I |
| 35. | CH-G4 microsatellite | KJ885221 | | India | 10 | II |
| 36. | CH-S3 microsatellite | KJ885220 | | India | 3 | I |
| 37. | CH-R1 microsatellite | KJ885219 | | India | 11 | III |
| 38. | AP7 microsatellite | KJ885190 | | India | 5 | I |
| 39. | AP2 microsatellite | KJ885189 | | India | 5 | I |
| 40. | AP9 microsatellite | KJ885188 | | India | 5 | I |
| 41. | AP6 microsatellite | KJ885187 | | India | 5 | I |
| 42. | TNal-12 microsatellite | KJ885254 | | India | 13 | III |
| 43. | TNal-11 microsatellite | KJ885253 | | India | 5 | I |
| 44. | TNal-10 microsatellite | KJ885252 | | India | 7 | II |
| 45. | TNal-9 microsatellite | KJ885251 | | India | 8 | II |
| 46. | TNal-8 microsatellite | KJ885250 | | India | 5 | I |
| 47. | TNal-7 microsatellite | KJ885249 | | India | 5 | I |
| 48. | TNal-6 microsatellite | KJ885248 | | India | 10 | II |
| 49. | TNal-5 microsatellite | KJ885247 | | India | 11 | III |
| 50. | TNal-4 microsatellite | KJ885246 | | India | 4 | I |
| 51. | TNal-3 microsatellite | KJ885245 | | India | 13 | III |
| 52. | TNal-2 microsatellite | KJ885244 | | India | 5 | I |
| 53. | P--3 microsatellite | KJ885255 | | India | 4 | I |
| 54. | AN-5 microsatellite | KJ921636 | | India | 6 | II |
| 55. | AN-4 microsatellite | KJ921635 | | India | 13 | III |
| 56. | AN-3 micsast | KJ921634 | | India | 6 | II |
| 57. | An microsatellite | KJ921633 | | India | 6 | II |
| 58. | A-3 microsatellite | KJ921632 | | India | 5 | I |
| 59. | Ex-2 microsatellite | KJ885226 | | India | 6 | II |
| 60. | Ex-1 microsatellite | KJ885225 | | India | 6 | II |
| 61. | R4P9 microsatellite | KJ885224 | | India | 5 | I |
| 62. | B35-120 microsatellite | KJ885218 | | India | 9 | II |
| 63. | B35-51 microsatellite | KJ885217 | | India | 5 | I |
| 64. | B35-13 microsatellite | KJ885216 | | India | 8 | II |
| 65. | B35-10 microsatellite | KJ885215 | | India | 5 | I |
| 66. | B35-7 microsatellite | KJ885214 | | India | 9 | II |
| 67. | IPB-127 microsatellite | KJ885213 | | India | 8 | II |
| 68. | IPB-118 microsatellite | KJ885212 | | India | 5 | I |
| 69. | IPB-66 microsatellite | KJ885211 | | India | 9 | II |
| 70. | IPB-49 microsatellite | KJ885210 | | India | 9 | II |
| 71. | IPB-24 microsatellite | KJ885209 | | India | 9 | II |
| 72. | MH-BL5P5 microsatellite | KJ885239 | | India | 16 | IV |
| 73. | MH-BL4P11 microsatellite | KJ885238 | | India | 12 | III |
| 74. | MH-BL3P25 microsatellite | KJ885237 | | India | 2 | I |
| 75. | MH-BL3P21 microsatellite | KJ885236 | | India | 17 | IV |
| 76. | MH-BL3P7 microsatellite | KJ885235 | | India | 13 | III |
| 77. | MH-BL3P4 microsatellite | KJ885234 | | India | 7 | II |
| 78. | MH-R-AL microsatellite | KJ885233 | | India | 9 | II |
| 79. | MH-R-RL microsatellite | KJ885232 | | India | 12 | III |
| 80. | MH-R-MO microsatellite | KJ885231 | | India | 9 | II |
| 81. | MH-R-CM microsatellite | KJ885230 | | India | 8 | II |
| 82. | MnH-1 microsatellite | MH781492 | | India | 4 | I |
| 83. | MnH-3 microsatellite | MH781493 | | India | 4 | I |
| 84. | MnH-7 microsatellite | MH781494 | | India | 4 | I |
| 85. | MnH-15 microsatellite | MH781495 | | India | 12 | III |
| 86. | MnH-19 microsatellite | MH781496 | | India | 20 | IV |
| 87. | Mnh-21 microsatellite | MH781497 | | India | 4 | I |
| 88. | Mnh-22 microsatellite | MH781498 | | India | 8 | II |
| 89. | Mnh-23 microsatellite | MH781499 | | India | 2 | I |
| 90. | Mnh-24 microsatellite | MH781500 | | India | 5 | I |
| 91. | Mnh-25 microsatellite | MH781501 | | India | 5 | I |
| 92. | Mnh-26 microsatellite | MH781502 | | India | 5 | I |
| 93. | Mnh-27 microsatellite | MH781503 | | India | 5 | I |
| 94. | Mnh-28 microsatellite | MH781504 | | India | 5 | I |
| 95. | Mnh-30 microsatellite | MH781505 | | India | 5 | I |
| 96. | Mnh-31 microsatellite | MH781506 | | India | 6 | II |
| 97. | Mnh-35 microsatellite | MH781507 | | India | 3 | I |
| 98. | Mnh-36 microsatellite | MH781508 | | India | 12 | III |
| 99. | Mnh-55 microsatellite | MH781509 | | India | 6 | II |
| 100. | Mnh-56 microsatellite | MH781510 | | India | 6 | II |
| 101. | Mnh-57 microsatellite | MH781511 | | India | 9 | II |
| 102. | Mnh-62 microsatellite | MH781512 | | India | 11 | III |
| 103. | Mnh-63 microsatellite | MH781513 | | India | 6 | II |
| 104. | Mnh-65 microsatellite | MH781514 | | India | 5 | I |
| 105. | Mnh-66 microsatellite | MH781515 | | India | 5 | I |
| 106. | Mnh-86 microsatellite | MH781516 | | India | 7 | II |
| 107. | Mnh-88 microsatellite | MH781517 | | India | 5 | I |
| 108. | Mnh-88 microsatellite | MH781518 | | India | 9 | II |
| 109. | Mnh-88 microsatellite | MH781519 | | India | 4 | I |
| 110. | Mnh-94 microsatellite | MH781520 | | India | 3 | I |
| 111. | Mnh-99 microsatellite | MH781521 | | India | 4 | I |
| 112. | Mnh-101 microsatellite | MH781522 | | India | 6 | II |
| 113. | Mnh-105 microsatellite | MH781523 | | India | 7 | II |
| 114. | Mnh-106 microsatellite | MH781524 | | India | 7 | II |
| 115. | Mnh-108 microsatellite | MH781525 | | India | 7 | II |
| 116. | NgH-1 microsatellite | MH781526 | | India | 5 | I |
| 117. | NgH-7 microsatellite | MH781527 | | India | 2 | I |
| 118. | NgH-9 microsatellite | MH781528 | | India | 8 | II |
| 119. | NgH- 10 microsatellite | MH781529 | | India | 8 | II |
| 120. | NgH-11 microsatellite | MH781530 | | India | 8 | II |
| 121. | NgH-12 microsatellite | MH781531 | | India | 5 | I |
| 122. | TrH-1 microsatellite | MH781532 | | India | 4 | I |
| 123. | TrH-2 microsatellite | MH781533 | | India | 9 | II |
| 124. | TrH-3 microsatellite | MH781534 | | India | 4 | I |
| 125. | TrH-4 microsatellite | MH781535 | | India | 9 | II |
| 126. | TrH-5 microsatellite | MH781536 | | India | 6 | II |
| 127. | TrH-6 microsatellite | MH781537 | | India | 6 | II |
| 128. | MgH-1 microsatellite | MH781538 | | India | 4 | I |
| 129. | MgH-2 microsatellite | MH781539 | | India | 6 | II |
| 130. | MgH-4 microsatellite | MH781540 | | India | 9 | II |
| 131. | MgH-5 microsatellite | MH781541 | | India | 7 | II |
| 132. | MgH-6 microsatellite | MH781542 | | India | 7 | II |
| 133. | AnH-5 microsatellite | MH781543 | | India | 8 | II |
| 134. | AnH-8 microsatellite | MH781544 | | India | 12 | III |
| 135. | AnH-12 microsatellite | MH781545 | | India | 9 | II |
| 136. | AnH-13 microsatellite | MH781546 | | India | 9 | II |
| 137. | CITNM09 microsatellite | [MW380106](https://www.ncbi.nlm.nih.gov/nucleotide/MW380106?report=genbank&log$=nucltop&blast_rank=1&RID=YPF73HBM013) | | India | 8 | II |
| 138. | CITMO015 microsatellite | [MW380102](https://www.ncbi.nlm.nih.gov/nucleotide/MW380102?report=genbank&log$=nucltop&blast_rank=2&RID=YPF73HBM013) | | India | 8 | II |
| 139. | CITMO02 microsatellite | [MW380104](https://www.ncbi.nlm.nih.gov/nucleotide/MW380104?report=genbank&log$=nucltop&blast_rank=3&RID=YPF73HBM013) | | India | 8 | II |
| 140. | CITAL05 microsatellite | MW380116 | | India | 7 | II |
| 141. | CITCM02 microsatellite | MW380089 | | India | 7 | II |
| 142. | CITNM25 microsatellite | [MW380101](https://www.ncbi.nlm.nih.gov/nucleotide/MW380101?report=genbank&log$=nucltop&blast_rank=6&RID=YPF73HBM013) | | India | 7 | II |
| 143. | CITNM26 microsatellite | [MW380096](https://www.ncbi.nlm.nih.gov/nucleotide/MW380096?report=genbank&log$=nucltop&blast_rank=7&RID=YPF73HBM013) | | India | 7 | II |
| 144. | CITMO07 microsatellite | [MW380108](https://www.ncbi.nlm.nih.gov/nucleotide/MW380108?report=genbank&log$=nucltop&blast_rank=8&RID=YPF73HBM013) | | India | 9 | II |
| 145. | CITP30 microsatellite | [MW380107](https://www.ncbi.nlm.nih.gov/nucleotide/MW380107?report=genbank&log$=nucltop&blast_rank=9&RID=YPF73HBM013) | | India | 9 | II |
| 146. | CITKhM29 microsatellite | [MW380091](https://www.ncbi.nlm.nih.gov/nucleotide/MW380091?report=genbank&log$=nucltop&blast_rank=10&RID=YPF73HBM013) | | India | 7 | II |
| 147. | CITNM66 microsatellite | [MW380109](https://www.ncbi.nlm.nih.gov/nucleotide/MW380109?report=genbank&log$=nucltop&blast_rank=11&RID=YPF73HBM013) | | India | 7 | II |
| 148. | CITPO21 microsatellite | MW380085 | | India | 7 | II |
| 149. | CITNM05 microsatellite | MW380092 | | India | 6 | II |
| 150. | CITKMP08 microsatellite | MZ128153 | | India | 8 | II |
| 151. | India_Jorhat_2 | OM728241 | | India | 6 | II |
| 152. | India_Jorhat_6 | OM728245 | | India | 6 | II |
| 153. | India_Jorhat_1 | OM728240 | | India | 6 | II |
| 154. | CITKMP26 microsatellite | MZ128152 | | India | 9 | II |
| 155. | CITCMK01 microsatellite | MZ128154 | | India | 9 | II |
| 156. | Inida_Punjab_2 | OM728230 | | India | 6 | II |
| 157. | India_Jorhat_5 | OM728244 | | India | 6 | II |
| 158. | CITKMA16 microsatellite | MZ128156 | | India | 7 | II |
| 159. | CITKMP12 microsatellite | MZ128151 | | India | 7 | II |
| 160. | CITNM16 microsatellite | MT747027 | | India | 6 | II |
| 161. | CITNMMP21 | MZ128157 | | India | 6 | II |
| 162. | CITDM05 | [MT747028.1](https://www.ncbi.nlm.nih.gov/nucleotide/F213MT747028.1?report=genbank&log$=nucltop&blast_rank=11&RID=ZZFXCZCB01N) | | India | 11 | III |
| 163. | CITKMA06 microsatellite | [MZ128155.1](https://www.ncbi.nlm.nih.gov/nucleotide/MZ128155.1?report=genbank&log$=nucltop&blast_rank=1&RID=ZZFXCZCB01N) | | India | 9 | II |
| 164. | CITP31 microsatellite | MT747026 | | India | 5 | II |
| 165. | CITDM06 microsatellite | MT747029 | | India | 11 | III |
| 166. | CITKMP08 microsatellite | [MZ128153.1](https://www.ncbi.nlm.nih.gov/nucleotide/MZ128153.1?report=genbank&log$=nucltop&blast_rank=4&RID=ZZFXCZCB01N) | | India | 8 | II |
| 167. | CGB-73 microsatellite | PQ505954 | | India | 4 | I |
| 168. | CGB-21 microsatellite | PQ505972 | | India | 4 | I |
| 169. | CGB-12 microsatellite | PQ505978 | | India | 9 | II |
| 170. | CGB-77 microsatellite | PQ505952.1 | | India | 2 | I |
| 171. | CITMO06 microsatellite | MW380103.1 | | India | 8 | II |
| 172. | CITKM16 microsatellite | MW380090 | | India | 11 | III |
| 173. | CITRL47 microsatellite | MW380093 | | India | 11 | III |
| 174. | CGB-25 microsatellite | PQ505970 | | India | 11 | III |
| 175. | CGB-88 microsatellite | PQ505946 | | India | 10 | II |
| 176. | CGB-142 microsatellite | PQ505923 | | India | 11 | III |
| 177. | CGB-94 microsatellite | PQ505943 | | India | 7 | II |
| 178. | SA.Nijran.52.19 | OK362284 | | Saudi Arabia | 4 | I |
| 179. | SA.Nijran.46.3 | OK362282 | | Saudi Arabia | 4 | I |
| 180. | SA.Nijran.46.2 | OK362281 | | Saudi Arabia | 4 | I |
| 181. | SA.Tabook.28.2 | OK362280 | | Saudi Arabia | 4 | I |
| 182. | SA.Nijran.52.15 | OK362279 | | Saudi Arabia | 4 | I |
| 183. | SA.Al-Qassim.43.1 | OK362278 | | Saudi Arabia | 4 | I |
| 184. | SA.Nijran.52.14 | OK362276 | | Saudi Arabia | 4 | I |
| 185. | SA.Nijran.48.10 | OK362270 | | Saudi Arabia | 4 | I |
| 186. | SA.Nijran.48.1 | OK362266 | | Saudi Arabia | 4 | I |
| 187. | SA.Jeddah.15.4 | OK362264 | | Saudi Arabia | 4 | I |
| 188. | SA.Jeddah.15.2 | OK362261 | | Saudi Arabia | 4 | I |
| 189. | SA.Nijran.52.17 | OK362260 | | Saudi Arabia | 4 | I |
| 190. | SA.Nijran.49.12 | OK362259 | | Saudi Arabia | 4 | I |
| 191. | SA.Jeddah.15.1 | OK362257 | | Saudi Arabia | 4 | I |
| 192. | SA.AL-ULA.76.1 | OK362242 | | Saudi Arabia | 4 | I |
| 193. | SA.AL-ULA.71.1 | OK362241 | | Saudi Arabia | 4 | I |
| 194. | SA.AL-ULA.68.8 | OK362240 | | Saudi Arabia | 4 | I |
| 195. | SA.HAIL.61.3 | OK362239 | | Saudi Arabia | 4 | I |
| 196. | SA.HAIL.60.5 | OK362238 | | Saudi Arabia | 4 | I |
| 197. | SA.HAIL.61.2 | OK362237 | | Saudi Arabia | 4 | I |
| 198. | SA.Nijran.52.13 | OK362283 | | Saudi Arabia | 5 | I |
| 199. | SA.Tabook.27.6 | OK362269 | | Saudi Arabia | 5 | I |
| 200. | SA.Nijran.52.5 | OK362267 | | Saudi Arabia | 5 | I |
| 201. | SA.Al-bahah.23.fr | OK362265 | | Saudi Arabia | 5 | I |
| 202. | SA.Al-bahah.23 | OK362262 | | Saudi Arabia | 5 | I |
| 203. | SA.Nijran.49.11 | OK362255 | | Saudi Arabia | 5 | I |
| 204. | SA.Jeddah.14.4 | OK362253 | | Saudi Arabia | 5 | I |
| 205. | SA.HAIL.60.3 | OK362236 | | Saudi Arabia | 5 | I |
| 206. | SA.HAIL.60.1 | OK362235 | | Saudi Arabia | 5 | I |
| 207. | SA.Tabook.N.2.3 | OK362272 | | Saudi Arabia | 5 | I |
| 208. | SA.Al-bahah.21.7 | OK362258 | | Saudi Arabia | 5 | I |
| 209. | SA.AL-ULA.76.7 | OK362244 | | Saudi Arabia | 5 | I |
| 210. | SA.HAIL.61.1 | OK362243 | | Saudi Arabia | 5 | I |
| 211. | SA.Al-bahah.21.2 | OK362273 | | Saudi Arabia | 2 | I |
| 212. | SA.Al-bahah.21.4 | OK362274 | | Saudi Arabia | 2 | I |
| 213. | SA.Al-bahah.21.4.fr | OK362277 | | Saudi Arabia | 2 | I |
| 214. | SA.Al-bahah.21.4 | OK362277 | | Saudi Arabia | 2 | I |
| 215. | Nep2 microsatellite | MG384795 | | Nepal | 6 | II |
| 216. | Nep3 microsatellite | MG384796 | | Nepal | 9 | II |
| 217. | Nep4 microsatellite | MG384797 | | Nepal | 8 | II |
| 218. | Bhu-Zh-71-L | MN127785 | | Nepal | 13 | III |
| 219. | Nep 5 microsatellite | MG384798 | | Nepal | 8 | II |
| 220. | Nep 6 microsatellite | MG384799 | | Nepal | 6 | II |
| 221. | Nep 7 microsatellite | MG384800 | | Nepal | 5 | I |
| 222. | Bhu-Sa-78-L | MN127786 | | Bhutan | 8 | II |
| 223. | Bhu-Sa-82-L | MN127787 | | Bhutan | 7 | II |
| 224. | Bhu-Da-76-L | MT592814 | | Bhutan | 7 | II |
| 225. | Bhu-Zh-68-L | MT592816 | | Bhutan | 12 | III |
| 226. | Bhu-Ts-82-L | MN127787 | | Bhutan | 7 | II |
| 227. | Bhu-Ts-1-L microsatellite | MN127782 | | Bhutan | 7 | II |
| 228. | Bhu-Ts-3-L microsatellite | MN127783 | | Bhutan | 7 | II |
| 229. | Bhu-Ts-20-L microsatellite | MT592817 | | Bhutan | 8 | II |
| 230. | Bhu-Ts-9-L microsatellite | MT592815 | | Bhutan | 7 | II |
| 231. | Bhu-da-36-L | MN127784 | | Bhutan | 8 | II |
| 232. | GZLD6 chromosome | CP131273.1 | | China | 6 | II |
| 233. | GDDQ6 chromosome | CP131387.1 | | China | 7 | II |
| 234. | FJNP3c chromosome | CP131428.1 | | China | 6 | II |
| 235. | YNBC chromosome | CP118771.1 | | China | 5 | I |
| 236. | GDSHH2 chromosome | CP131327.1 | | China | 6 | II |
| 237. | GDCH12 chromosome | CP159585 | | China | 4 | I |
| 238. | PY chromosome | CP100417 | | China | 5 | I |
| 239. | GDCZ2JI chromosome | CP131401 | | China | 7 | II |
| 240. | GDCZS3A4 chromosome | CP131393 | | China | 7 | II |
| 241. | GDDQ7 chromosome | CP131384 | | China | 7 | II |
| 242. | GDHYHP4a chromosome | CP131375 | | China | 7 | II |
| 243. | GDHZ11 chromosome | CP131370 | | China | 7 | II |
| 244. | GDHZ13 chromosome | CP131367 | | China | 7 | II |
| 245. | GDNXA11 chromosome | CP131339 | | China | 7 | II |
| 246. | GDXH2 chromosome | CP131319 | | China | 7 | II |
| 247. | GDXH9661 chromosome | CP131317 | | China | 7 | II |
| 248. | GDYJ14 chromosome | CP131313 | | China | 7 | II |
| 249. | GXLP2 chromosome | CP131301 | | China | 7 | II |
| 250. | GZLD2 chromosome | CP131277 | | China | 7 | II |
| 251. | HUNJY16 chromosome | CP131236 | | China | 7 | II |
| 252. | HUNYX16 chromosome | CP131234 | | China | 7 | II |
| 253. | SCXCQC7 chromosome | CP131194 | | China | 7 | II |
| 254. | YNJS3QI chromosome | CP131176 | | China | 7 | II |
| 255. | YNJS3W chromosome | CP131174 | | China | 7 | II |
| 256. | YNJS9352 chromosome | CP131162 | | China | 7 | II |
| 257. | ZJTZ7 chromosome | CP131134 | | China | 7 | II |
| 258. | GDCZ chromosome | CP118922 | | China | 7 | II |
| 259. | GDMM26 chromosome | CP131352 | | China | 7 | II |
| 260. | GDCZPG5 chromosome | CP131395 | | China | 8 | II |
| 261. | GDHY1 chromosome | CP131380 | | China | 8 | II |
| 262. | HUNYZ7 chromosome | CP131228 | | China | 8 | II |
| 263. | JXGN13 chromosome | CP131223 | | China | 8 | II |
| 264. | JXGN8 chromosome | CP131220 | | China | 8 | II |
| 265. | YNJSMGD chromosome | CP131169 | | China | 8 | II |
| 266. | YNRL13 chromosome | CP131145 | | China | 8 | II |
| 267. | YNSL128 chromosome | CP131175 | | China | 8 | II |
| 268. | YNDL8465 chromosome | CP131185 | | China | 6 | II |
| 269. | ZJHY8 chromosome | CP131143 | | China | 6 | II |
| 270. | GZQL4 chromosome | CP124118 | | China | 6 | II |
| 271. | FJFQ1 chromosome | CP131438 | | China | 6 | II |
| 272. | FJMHLG9 chromosome | CP131430 | | China | 6 | II |
| 273. | FJQZ7992 chromosome | CP131426 | | China | 6 | II |
| 274. | FJSMDT7 chromosome | CP131414 | | China | 6 | II |
| 275. | FJYC1L chromosome | CP131412 | | China | 6 | II |
| 276. | GDMZ2 chromosome | CP131343 | | China | 5 | I |
| 277. | GDDG8 chromosome | CP131391 | | China | 6 | II |
| 278. | GDGZ1981 chromosome | CP131382 | | China | 6 | II |
| 279. | GDLZX2F chromosome | CP131359 | | China | 6 | II |
| 280. | GDHZF3P chromosome | CP131361 | | China | 6 | II |
| 281. | GDMMLM1 | CP131349 | | China | 6 | II |
| 282. | GDNXB6 chromosome | CP131337 | | China | 6 | II |
| 283. | GDQY8 chromosome | CP131335 | | China | 6 | II |
| 284. | GDCZ8042 chromosome | CP131399 | | China | 6 | II |
| 285. | HANQZ5 chromosome | CP131240 | | China | 6 | II |
| 286. | FJNP3c chromosome | CP131428 | | China | 6 | II |
| 287. | GDSG8240 chromosome | CP131331 | | China | 6 | II |
| 288. | GXRX51 chromosome | CP131291 | | China | 6 | II |
| 289. | ZJTZ32 chromosome | CP131137 | | China | 6 | II |
| 290. | YNGNLG2 chromosome | CP131183 | | China | 6 | II |
| 291. | GZCJ1 chromosome | CP131281 | | China | 6 | II |
| 292. | SCYB13F chromosome | CP131192 | | China | 6 | II |
| 293. | GDSG8267 chromosome | CP131329 | | China | 6 | II |
| 294. | YNJS7 chromosome | CP131178 | | China | 6 | II |
| 295. | YNJSMGG chromosome | CP132149 | | China | 6 | II |
| 296. | SCXCQC39 | CP131199 | | China | 6 | II |
| 297. | HUNYZ10 chromosome | CP131230 | | China | 6 | II |
| 298. | GDQYA6151 | CP131333 | | China | 6 | II |
| 299. | YNRLNJ5 chromosome | CP131148 | | China | 6 | II |
| 300. | ZJHY28 chromosome | CP131155 | | China | 6 | II |
| 301. | ZJTZ1502 chromosome | CP131139 | | China | 6 | II |
| 302. | YNJS9347 chromosome | CP131164 | | China | 6 | II |
| 303. | JXGZ8421 chromosome | CP131217 | | China | 6 | II |
| 304. | HANCMY14F | CP131265 | | China | 6 | II |
| 305. | FJYC3 chromosome | CP131410 | | China | 6 | II |
| 306. | GDSQ3460 chromosome | CP131323 | | China | 6 | II |
| 307. | YNGNMG1 | CP131181 | | China | 6 | II |
| 308. | ZJOH1 chromosome | CP131141 | | China | 6 | II |
| 309. | GDSQFS73 chromosome | CP131321 | | China | 6 | II |
| 310. | YNXP3P chromosome | CP131157 | | China | 6 | II |
| 311. | YNNL8506 chromosome | CP131152 | | China | 6 | II |
| 312. | GXNN34 chromosome | CP131295 | | China | 6 | II |
| 313. | SCDCSTJ18 | CP131210 | | China | 6 | II |
| 314. | GDZC10 chromosome | CP131309 | | China | 6 | II |
| 315. | GDYF2159 chromosome | CP131315 | | China | 6 | II |
| 316. | YNLJ2Fe chromosome | CP131150 | | China | 6 | II |
| 317. | GZCJ2 chromosome | CP131279 | | China | 6 | II |
| 318. | HUNBZ8153 | CP131238 | | China | 6 | II |
| 319. | HANQZ4a chromosome | CP131242 | | China | 10 | II |
| 320. | HANQZ3d chromosome | CP131246 | | China | 10 | II |
| 321. | SCNN6 chromosome | CP131201 | | China | 9 | II |
| 322. | HANQHF1 chromosome | CP131256 | | China | 9 | II |
| 323. | GDCZJG9 chromosome | CP131397 | | China | 9 | II |
| 324. | GDHZ3 chromosome | CP131365 | | China | 16 | IV |
| 325. | GDZJ27 chromosome | CP131307 | | China | 16 | IV |
| 326. | FJMHLG6 chromosome | CP131432 | | China | 18 | IV |
| 327. | SCLBGM5c | CP131205 | | China | 11 | III |
| 328. | GDZJ6 chromosome | CP131305 | | China | 11 | III |
| 329. | GDHZ10 chromosome | CP131372 | | China | 11 | III |
| 330. | SCXCQC6 chromosome | CP131196 | | China | 12 | III |
| 331. | HANDZ6 chromosome | CP131258 | | China | 12 | III |
| 332. | GXYF4 chromosome | CP131285 | | China | 12 | III |
| 333. | FJSC2d chromosome | CP131421 | | China | 5 | I |
| 334. | PGD chromosome | CP100754 | | China | 6 | II |
| 335. | FJSMDT5 chromosome | CP131416 | | China | 11 | III |
| 336. | GDCH1012 | CP131408 | | China | 4 | I |
| 337. | GDHYHP3 chromosome | CP131377 | | China | 9 | II |
| 338. | GDYJ151 chromosome | CP131311 | | China | 4 | I |
| 339. | GDHZ4 chromosome | CP131363 | | China | 3 | I |
| 340. | Str Gxpsy | CP004005 | | China | 8 | II |
| 341. | GDHZ11D chromosome | CP045565 | | China | 7 | II |
| 342. | Strain A4, whole genome | CP010804 | | China | 6 | II |
| 343. | JXGC chromosome | CP019958 | | China | 6 | II |
| 344. | PGD chromosome | CP100754 | | China | 6 | II |
